# Results From a Phase 2, Open‐Label Study Evaluating the Safety, Tolerability, and Effect on Ataxia of GLM101 in Three Adult Patients With PMM2‐CDG

**DOI:** 10.1002/jimd.70240

**Published:** 2026-09-07

**Authors:** Mercedes Serrano, Florencia Epifani, Rose Marino, Peter McWilliams

**Affiliations:** ^1^ Neuropediatric Department, Hospital Sant Joan de Déu Institut de Recerca Sant Joan de Déu (IRSJD) Barcelona Spain; ^2^ U‐703 Center for Biomedical Research Network on Rare Diseases (CIBERER) Instituto de Salud Carlos III (ISCIII) Madrid Spain; ^3^ Universitat de Barcelona Barcelona Spain; ^4^ Glycomine Inc. San Carlos California USA

**Keywords:** ataxia, cerebellar syndrome, GLM101, ICARS, phosphomannomutase deficiency, PMM2‐CDG

## Abstract

Phosphomannomutase 2 congenital disorder of glycosylation (PMM2‐CDG) is a rare, autosomal recessive disease caused by PMM2 deficiency, which impairs conversion of mannose‐6‐phosphate into mannose‐1‐phosphate (M1P) and disrupts N‐linked glycosylation. It typically results in a multisystem disorder in which a prominent cerebellar syndrome, characterized by ataxia among other clinical manifestations, can be assessed using the International Cooperative Ataxia Rating Scale (ICARS). GLM101 is a liposomal M1P substrate replacement therapy in clinical development. We present results conducted both within and outside the protocol‐defined schedule from three adult patients enrolled in a Phase 2 study evaluating the efficacy, safety, and tolerability of GLM101 (NCT05549219). PMM2‐CDG patients received weekly infusions of 30 mg/kg GLM101 for 24 weeks. Ataxia was measured by ICARS as standard of care. Absolute and percent change from baseline were calculated. Additional results reported include global impression of change scales (caregiver and clinician) and safety. Three adult patients (1 M, 2 F) completed 24 weeks of treatment. The mean (SD) baseline ICARS was 50.7 (19.0) and mean (SD) change from baseline was −14.0 (7.2) and −17.7 (3.1) at Weeks 12 and 24, respectively. Improvements were seen across all ICARS subdomains. All patients and clinicians reported global clinical improvement. GLM101 was well tolerated with no serious adverse events. Infusion‐associated reactions were reported in one patient, with no need to interrupt the therapy. Safety findings showed no adverse trends. In conclusion, GLM101 was well tolerated and demonstrated potential for meaningful clinical benefit. These findings support continued evaluation of GLM101 in patients with PMM2‐CDG.

## 1 Introduction

Phosphomannomutase 2 congenital disorder of glycosylation (PMM2‐CDG) is a rare, inherited autosomal recessive metabolic disease affecting protein glycosylation [1]. PMM2‐CDG is caused by a deficiency in PMM2, the enzyme that converts mannose‐6‐phosphate into mannose‐1‐phosphate (M1P), resulting in unoccupied N‐linked glycosylation sites or altered glycan structures on a wide range of proteins [1]. The clinical phenotype is highly variable, ranging from mild forms with minimal organ involvement to severe forms resulting in early mortality [2]. Approximately 12% of infants with PMM2‐CDG do not survive the first year and 20% do not survive the fifth year due to multiple organ failure [3]. Early clinical features typically include abnormal fat distribution, inverted nipples, strabismus, and hypotonia [2, 4]. Affected infants frequently develop ataxia, psychomotor delay, and a range of nonneurological symptoms, such as failure to thrive, protein‐losing enteropathy, liver failure, cardiomyopathy, retinopathy, coagulopathies, and cardiac and renal abnormalities [5]. Recent studies have highlighted the importance patients, caregivers, family members, and treating physicians place on addressing persistent neurological symptoms, which significantly impact patients' functionality, autonomy, and quality of life [6].

While in CDG an abnormal sialotransferrin profile, a consequence of defective N‐glycosylation, lends support to the diagnosis, it is not a useful biomarker for stratification or monitoring, as it can show spontaneous improvement with age [7]. Despite the advent of advanced technologies enabling the identification of diverse molecular and functional markers, no laboratory biomarker has yet been clearly correlated with clinical presentation, disease severity, or progression—an important unmet need that would greatly aid the objective evaluation of patient benefits and outcomes. However, even in the absence of validated biological biomarkers, families consistently emphasize functional abilities as the most meaningful indicators of improvement and quality of life. In this sense, nearly all patients with PMM2‐CDG experience cerebellar atrophy, which leads to a cerebellar syndrome characterized as axial and peripheral ataxia, abnormal eye movements, dysarthria, and cognitive deficits, and is a key driver of disease burden [6]. Ataxia severity in PMM2‐CDG correlates with severe functional disability [8, 9]. Current treatment guidelines recognize ICARS as the established standard of care for the assessment of ataxia in patients with PMM2‐CDG [8, 10]. Furthermore, an observational, cross‐sectional pediatric study comparing PMM2‐CDG patients and healthy controls demonstrated that International Cooperative Ataxia Rating Scale (ICARS) is a reliable instrument, with minimal inter‐rater variability [11]. Another study, averaging 20 months, showed stabilization or mild improvement in cerebellar function in PMM2‐CDG pediatric patients despite cerebellar volume loss [8]. Since the natural history of the progression of ataxia in adults and pediatrics with PMM2‐CDG has not been established, the prospective collection of such data would be important to further characterize this. Other diseases with ataxia as a clinical symptom, including Friedreich's ataxia [12] and multiple sclerosis [13], have also used ICARS as a validated assessment.

There are no currently approved therapies for PMM2‐CDG. Supplementation with mannose, either orally or continuously via IV administration, has been shown to be ineffective [7, 14]. Current treatment relies on nonstandardized, palliative measures to manage clinical manifestations. GLM101 is a liposomal formulation encapsulating the active pharmaceutical agent M1P inside the aqueous core of the lipid nanoparticles [15]. Encapsulation is designed to protect M1P from rapid degradation in the systemic circulation and enable cellular internalization.

Here, we present ICARS conducted outside the original protocol‐defined schedule as well as global impression of change (GIC) and safety evaluations within the scope of the protocol from three adult participants receiving GLM101 at 30 mg/kg weekly in an open label Phase 2 study evaluating the efficacy, safety, and tolerability of GLM101 in adult, adolescent, and pediatric patients with PMM2‐CDG (NCT05549219).

## 2 Methods

### 2.1 Study Population

Three adult patients are presented (aged 18, 40, and 42 years) with a molecularly confirmed diagnosis of PMM2‐CDG. All patients described here were enrolled and treated in Study NCT05549219; the clinical study identifiers for these three patients are 204–2007, 204–2008, and 204–2009, henceforth referred to as S01, S02, and S03, respectively. Subjects S01 (F, 42) and S02 (M, 40) were siblings (Table 1). As per the protocol, PMM2‐CDG diagnosis was identified through clinical signs and confirmed by biallelic pathogenic and/or likely pathogenic variants (S01 and S02 with the p.Arg141His/p.Thr237Met genotype and S03 with p.Phe157Ser/p.Arg162Trp). For variants of uncertain pathogenicity, demonstration of bi‐allelic variants and PMM2 enzyme activity consistent with a diagnosis of PMM2‐CDG was required. According to the study protocol, none of the participants had a diagnosis of a congenital disorder of glycosylation other than PMM2‐CDG, nor did they have another genetic condition that might alter the assessment or confer risk to the patient.

**TABLE 1:** Baseline demographics and clinical characteristics.

Patient ID	Age	Sex	Origin	Baseline ICARS
S01	42	F	Spain	52
S02	40	M	Spain	69
S03^a^	18	F	Spain	31^a^

Abbreviation: ICARS, International Cooperative Ataxia Rating Scale.

^a^ ICARS assessments 8 and 9 for S03 were performed incorrectly at baseline (sitting instead of supine) and were therefore excluded from total ICARS scores for all subsequent visits; this affected the absolute scores but did not impact evaluation of change over time.

During earlier protocol phases of study NCT05549219, participants were assigned to the different dose cohorts according to the predefined dose‐escalation design, with random allocation to dose cohorts where applicable. Thus, previous stages of this Phase 2 study included adult patients receiving GLM101 at 10 or 20 mg/kg/day. The three adult patients included in this report represent the first participants treated with GLM101 at 30 mg/kg/day for 24 weeks.

### 2.2 Study Design

This Phase 2 study was conducted after obtaining ethical approval from the Research Ethics Committee of the SJD Research Foundation and the Spanish Agency of Medicines and Medical Devices (AEMPS), as detailed in the corresponding section. Each participant provided written informed consent for participation in the Phase 2 study, as all participants were considered capable of providing informed consent from the perspective of their families, the medical team, and from a legal standpoint. However, family members and caregivers were present during the information process and provided support in explaining the study information when needed.

Patients received a weekly IV infusion of GLM101, at 30 mg/kg for 24 weeks.

Prophylactic fexofenadine was administered approximately 12 (±2) hours and 3 (±1) hours before GLM101 infusion to minimize the potential for infusion‐associated reactions (IARs). GLM101 was administered at an increasing rate of infusion, with the total administration time lasting approximately 4 h. All participants were closely monitored for safety during and after each GLM101 infusion. Comprehensive laboratory tests and complementary examinations were performed in accordance with international guidelines [10]. After completing the 24‐week treatment period, each participant entered a follow‐up period for evaluation of safety. The final follow‐up visit was conducted 4 weeks after the last infusion of GLM101.

### 2.3 Outcomes

In the initial protocol, the primary efficacy outcome was defined as changes from baseline in coagulation and antithrombotic parameters. Following subsequent protocol amendments, assessment of ataxia severity using the ICARS was incorporated as an exploratory endpoint in August 2023 based on clinical observations made during the study and as part of standard‐of‐care evaluations. Subsequently, ICARS was designated as an additional primary efficacy outcome since November 2026, while changes in coagulation and antithrombotic parameters remained co‐primary efficacy outcomes. The ICARS was performed prior to treatment with GLM101 (baseline) and after 12 and 24 weeks of treatment, as well as approximately 4 weeks after stopping treatment. The ICARS is scored out of 100 points with 19 total items encompassing four subdomains: specifically posture and gait disturbances (items 1–7, 34/100), limb kinetic function (items 8–14, 52/100), speech disorders (items 15–16, 8/100), and oculomotor disorders (items 17–19, 6/100) [11, 16]. Higher scores indicate higher levels of impairment. The ICARS assessments were performed by the same raters (F.E. and M.S.) throughout the study and were video‐recorded for subsequent review. Data, including raw values and percentages for each subdomain, are presented for all three patients.

Exploratory outcomes included patient/caregiver‐reported outcomes, such as the patient/caregiver and clinician GIC. It evaluates how much the patient's illness had improved or worsened relative to the baseline prior to the intervention. It was measured on a 7‐point scale with the lowest score (1) indicating the greatest improvement, the highest score (7) indicating the greatest worsening, and a score of 4 indicating no change.

Safety outcomes included adverse events (AEs), adverse events of special interest (AESIs), including IARs, severe adverse events (SAEs), deaths, discontinuations due to AEs, clinical laboratory tests, electrocardiogram, vital signs, and physical examination findings.

### 2.4 Statistical Analyses

No prospective calculations of statistical power were made. The sample size reflects the number of adult patients included in the analyses and provides exploratory efficacy information on ataxia (ICARS) assessed outside of the original protocol‐defined schedule, together with the safety and tolerability of multiple doses of GLM101 in adult patients with PMM2‐CDG. Analyses were descriptive.

## 3 Results

Three adult patients (aged 18, 40, and 42 years) from a single clinical site (Hospital Sant Joan de Déu, Barcelona, Spain) treated with GLM101 at the 30 mg/kg dose are included in this analysis. The patients were comprised of one male (40 years of age) and two females (18 and 42 years of age). All patients were of white and European ancestry and were recruited in Spain (Table 1).

**FIGURE 1:**
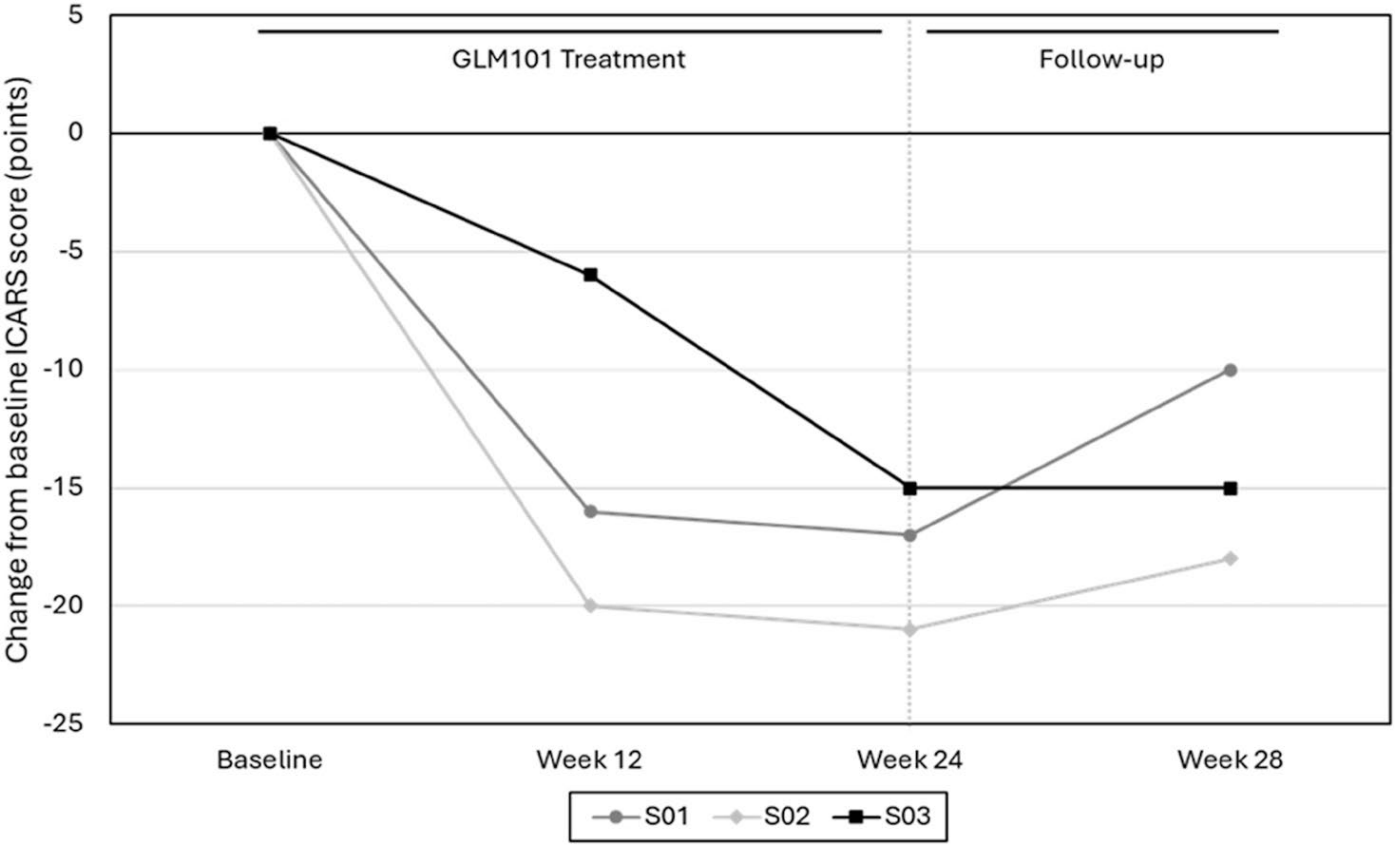
Change from baseline in total ICARS score. Change from baseline in International Cooperative Ataxia Rating Scale (ICARS) scores over time during GLM101 treatment and follow‐up. Data are shown for three individual participants (S01, S02, and S03). Assessments were performed at baseline, Weeks 12, 24 (end of treatment), and 28 (follow‐up). Negative values indicate improvement in ataxia severity.

**FIGURE 2:**
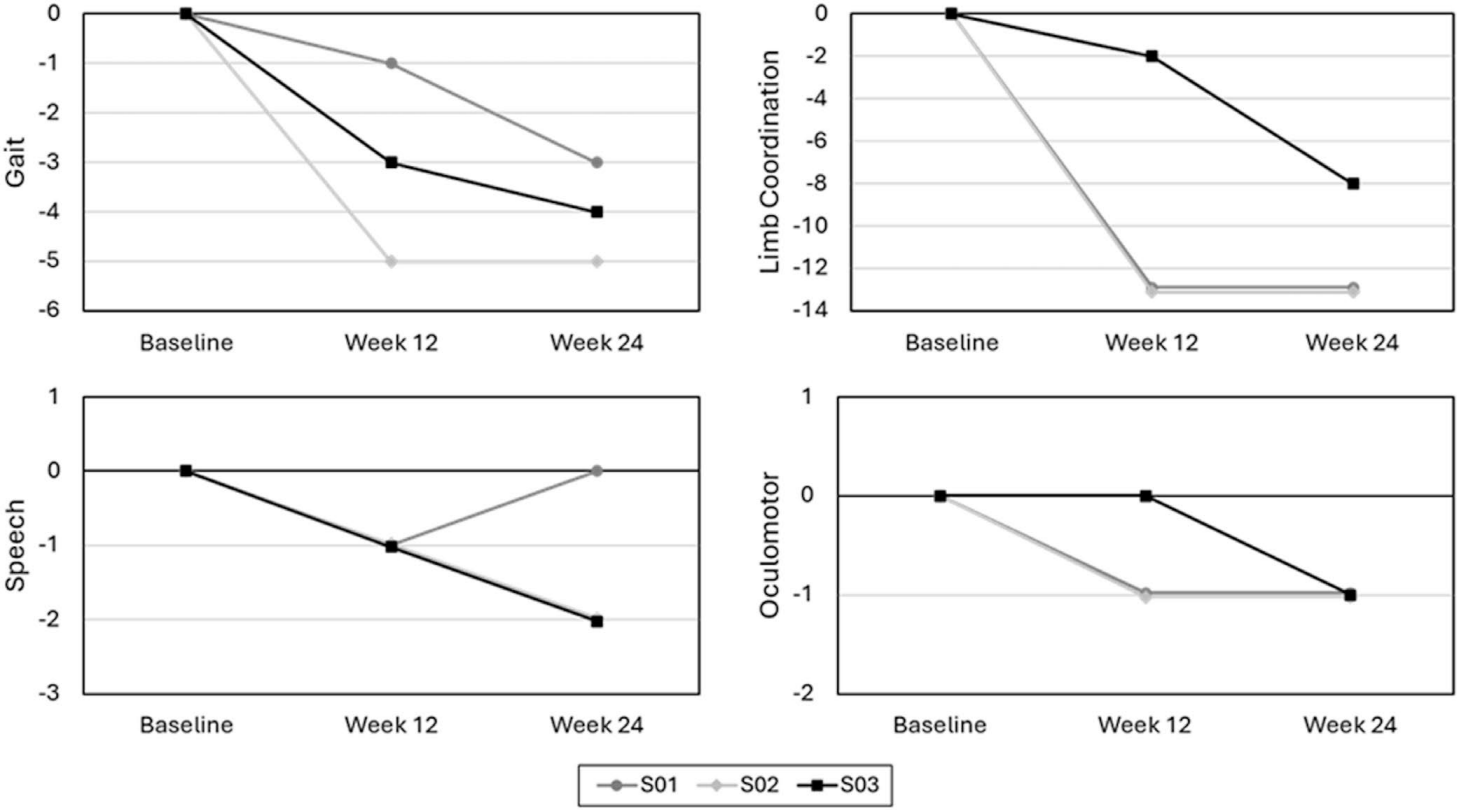
Change in ICARS sub‐domain scores. Change from baseline in International Cooperative Ataxia Rating Scale (ICARS) scores over time during GLM101 treatment and follow‐up, showing results across the different subdomains, with greater improvement in the limb kinetic function subdomain.

The mean (standard deviation, SD) change from baseline in ICARS was −14.0 (5.9) and −17.7 (2.5) at Weeks 12 and 24, respectively (Figure 1 and Table 2). The average ICARS subdomain improvement at Week 24 was −4.0 (17.7 at baseline) for posture and gait disturbances, −11.3 (26.3 at baseline) for limb kinetic function, −1.3 (4.0 at baseline) for speech disorders, and −1.0 (2.7 at baseline) for oculomotor disorders (Figure 2, Table 3, and Supporting Information), indicating that improvements were seen across all domains of the ICARS. Interestingly, at the follow‐up visit at Week 28, after discontinuation of the therapy, the mean change from baseline in ICARS was −14.3, with two of three patients showing a trend toward returning to baseline and one patient remaining stable. As ICARS was only reassessed at Week 28, the exact onset of deterioration cannot be determined. However, around 3 weeks after treatment discontinuation, two patients and their families reported increased hand tremor, instability, and a slight reduction in strength, consistent with the findings at the Week 28 evaluation. Specifically, deterioration was seen in both the posture and gait disturbances and limb kinetic function in both patients that exhibited this return toward baseline, while speech and oculomotor subscales remained stable. Nevertheless, the impact upon ICARS clearly seemed to diminish after therapy was withdrawn.

**TABLE 2:** Change from baseline in total ICARS score at Weeks 12, 24, and 28 (4 weeks follow‐up).

Patient ID	Baseline ICARS		Week 12 ICARS		Week 24 ICARS		Week 28 ICARS	
	Value	Change	Value	Change	Value	Change	Value	Change
S01	52	—	36	−16	35	−17	42	−10
S02	69	—	49	−20	48	−21	51	−18
S03^a^	31	—	25	−6	16	−15	16	−15
Mean (SD)	50.7 (19.0)	—	36.7 (12.0)	−14.0 (7.2)	33.0 (16.1)	−17.7 (3.1)	36.3 (18.1)	−14.3 (4.0)

Abbreviation: ICARS, International Cooperative Ataxia Rating Scale.

^a^ ICARS assessments 8 and 9 for S03 were performed incorrectly at baseline (sitting instead of supine) and were therefore excluded from total ICARS scores for all subsequent visits; this affected the absolute scores but did not impact evaluation of change over time.

**TABLE 3:** Mean (SD) change in ICARS sub‐domain scores at Week 24.

ICARS sub‐domain	Total potential ICARS	Mean (SD) baseline	Mean (SD) point change	Mean (SD) % change^a^
Posture and gait	34	17.7 (9.9)	−4.0 (1.0)	−25.6% (9.8%)
Limb Coordination	52	26.3 (9.8)	−11.3 (2.9)	−44.9% (7.3%)
Speech	8	4.0 (1.0)	−1.3 (1.2)	−35.6% (33.6%)
Oculomotor	6	2.7 (0.6)	−1.0 (0.0)	−38.9% (9.6%)

Abbreviation: ICARS, International Cooperative Ataxia Rating Scale.

^a^ Average of the percent changes for the individual subjects represents the mean of the individual percentage changes calculated for each subject, rather than the percentage change calculated from the pooled baseline and follow‐up values. Therefore, each patient contributes equally to the calculation, regardless of the absolute baseline values.

The overall percent improvement in ICARS was 34.9%, with the greatest changes seen in limb kinetic function (45%), followed by oculomotor (39%) and speech (36%) subdomains. The posture and gait subdomain was reduced by 26%, with some evidence of a ceiling effect; the most severe patient (baseline ICARS of 69) had a baseline score of 29/34 on the posture and gait subdomain, that included a score of 7/8 on item 1 (“walking only with an accompanying person”) and, subsequently, required to have the maximal score of 4/4 on item 2 (“walking with autonomous support no longer possible”) throughout the study. Of the three patients, each showed an improvement in every single domain of the ICARS except for one patient (S01), in which the speech subdomain remained stable. For one patient (S03), four items in the limb kinetic function were excluded (imputed to show no change), because the baseline measurements were recorded incorrectly; consequently, the changes seen in the ICARS for this patient are possibly underrepresented in this study. Table S1 provides item level ICARS scores at baseline, Weeks 12 and 24.

The mean (SD) patient/caregiver and clinician GIC at Week 24 were 1.7 (0.9) and 1.3 (0.5), respectively (Table 4). All patients/caregivers (three of three) and clinicians reported improvement in the GIC and in two of three, the patient/caregiver assessment agreed with the clinician assessment. Notably, clinicians and families reported perceived improvements beyond what is captured by the ICARS, such as attention, communication, and overall functionality.

**TABLE 4:** Patient/caregiver and clinician global impression of change at Week 24.

Patient ID	Patient/caregiver global impression of change^a^	Clinician global impression of change
S01	1 (very much improved)	1 (very much improved)
S02	1 (very much improved)	1 (very much improved)
S03	3 (minimally improved)	2 (much improved)
Mean (SD)	1.7 (0.9)	1.3 (0.5)

^a^ Global Impression of Change measures on a 7‐point scale with the lowest score (1) indicating the greatest improvement, the highest score (7) indicating the greatest worsening, and a score of 4 indicating no change.

GLM101 was well tolerated with no SAEs; only mild to moderate AEs were reported (Table 5). One patient reported treatment‐related AEs of intermittent hyperlipidemia (mild in severity) and pruritus (moderate in severity). One patient reported an AESI (IAR), which was moderate in severity with associated AEs of abdominal pain, vomiting, and conjunctival hyperemia. These were initially managed with methylprednisolone at 1 mg/kg, followed by a slow taper over the first 3 weeks (Table 5). No trends were observed in safety laboratory parameters (including blood cell counts, liver and kidney function, coagulation, electrolytes, and hormone levels), vital signs, physical examinations, or electrocardiograms. The coagulation factors that were abnormal at baseline remained essentially unchanged throughout the study. All the patients completed the 24 weeks of therapy, and none had to stop the therapy before the end of the treatment period.

**TABLE 5:** Summary of adverse events.

(a). Number of participants (%) by maximum toxicity grade	Grade 1: mild	Grade 2: moderate	Grade 3
Any adverse event (AE)	0	3 (100%)	0
Any treatment‐emergent AE	0	3 (100%)	0
Any treatment‐related AE	1 (33%)	1 (33%)	0
Any infusion‐associated reaction AE	0	1 (33%)	0

(b). Treatment‐related adverse event	*N* (%)	Grade	IAR related
Intermittent hyperlipidemia	1 (33%)	Grade 1: mild	No
Intermittent pruritus	1 (33%)	Grade 2: moderate	No
Abdominal pain	1 (33%)	Grade 2: moderate	Yes
Conjunctival hyperemia	1 (33%)	Grade 2: moderate	Yes
Vomiting	1 (33%)	Grade 2: moderate	Yes

Abbreviations: AE: adverse event; IAR: infusion‐associated reaction.

## 4 Discussion

In this study, we evaluated the safety, tolerability, and potential clinical effects of GLM101 administered over a 24‐week period in patients with PMM2‐CDG. Given that PMM2‐CDG is a multisystem medically complex disorder, participants were closely monitored for safety, while neurological function was also closely assessed, as it represents one of the main contributors to long‐term disability. To this end, the ICARS was used to explore whether GLM101 could provide measurable clinical benefit.

Treatment with GLM101 for 24 weeks was well tolerated, with only mild‐to‐moderate AEs and no SAEs or treatment discontinuations. All patients remained adherent to therapy. The single IAR (moderate IAR) was effectively managed with brief methylprednisolone, and isolated pruritus responded well to standard care. Safety parameters remained stable throughout, supporting GLM101's feasibility for longer‐term studies in this vulnerable population.

Regarding efficacy, a notable improvement in the ICARS score and its subdomains was observed, with the greatest change in limb kinetic function, suggesting a potentially meaningful clinical benefit in patients with PMM2‐CDG. Furthermore, in two of three patients, improvements were seen across all four subdomains of the ICARS and in the remaining patient, all subdomains except for one (that remained stable) improved. This broad improvement is particularly relevant given that PMM2‐CDG patients have broad ataxia involvement, including dysmetria, tremor, abnormal eye movements, and dysarthria, that contribute to long‐term disability [6, 8, 11]. Importantly, despite the small sample size (*n* = 3), clinical improvements were observed across patients with different degrees of neurological impairment at baseline, suggesting that the potential benefit of GLM101 may extend across severity ranges. Additionally, both patient/caregiver and clinician GIC reported improvement over the 24 week treatment period which supports the perceived improvements beyond what is captured by the ICARS, such as attention, communication, and overall functionality.

This study has several limitations and challenges that should be considered. First, the analysis was conducted in a relatively small subset of participants from a larger study, which limits the precision of the estimated effects and the generalizability of the findings. Second, the primary endpoint was amended during the course of the study. Specifically, study GLM101‐002 was initially designed around pharmacodynamic coagulation biomarkers, but the focus was later adjusted mid‐trial toward ataxia after improvements in ICARS were observed in the three participants discussed here. Given that ataxia is the most common and clinically relevant constellation of symptoms in PMM2‐CDG and can be assessed using ICARS, the primary endpoint was modified to better capture these findings. This scale has been previously demonstrated to be reliable for assessing ataxia in PMM2‐CDG patients [11] and includes subdomains for posture and gait, kinetic functions, speech abnormalities, and oculomotor function, all of which capture the features of ataxia in PMM2‐CDG.

Regarding challenges, administering ICARS can be difficult when assessing speech and oculomotor disorders in PMM2‐CDG, as previously reported [11], due to subjectivity and the influence of patient collaboration and understanding, which may hamper proper scoring and data interpretation. Therefore, it is extremely important that trained personnel administer the scale so that a reliable score can be obtained. Additionally, it is important to note that ICARS scores improve with age, in a nonlinear fashion, in healthy children [17]. These findings suggest that pediatric ICARS improvement may be age‐dependent although the natural history of ataxia progression in adults and pediatrics has not been established [8]. Overall, high interobserver reliability and correlation with other assessments support its use for evaluating ataxia severity in PMM2‐CDG [11, 18, 19]. Despite the utility of ICARS, the results do not necessarily follow a linear correlation with disease severity. Notably, some items within the posture and gait subscale are interdependent, and the scoring shows a ceiling effect, limiting its ability to differentiate patients with more severe ataxia. For example, inability to walk independently (score of 4–8 on item 1) automatically leads to maximum scores in related items assessing gait and balance. In other words, varying degrees of severity assessed in items 1 and 3 always result in a combined total of 16 points across items 2, 4, 5, and 6. Consequently, even when clinical improvements are observed, they may not be fully reflected in the posture and gait subscale. During data analysis, particular attention should be given to this ceiling effect observed within the posture and gait subscale.

Other shorter assessment tools, such as the Scale for the Assessment and Rating of Ataxia (SARA) [20], have limitations compared with ICARS. SARA does not assess oculomotor dysfunction, a relevant feature of PMM2‐CDG, and some tasks require adequate visual acuity, which may be impaired in these patients. Additionally, timed tasks may be influenced by the reduced processing speed and executive dysfunction commonly observed in cerebellar disorders, including PMM2‐CDG [21], potentially limiting the ability of SARA to capture meaningful clinical improvements.

In this cohort, the improvement observed in ataxia, as assessed by the ICARS, may also reflect secondary effects beyond cerebellar function, such as enhanced muscle tone and strength, given that myopathy and peripheral neuropathy have been reported in PMM2‐CDG [22]. Improvements in attention and performance capacity, reported by both families and clinicians, may have also contributed, although these effects could not be formally assessed. Despite the difficulty in distinguishing cerebellar‐specific effects from these concomitant factors, the overall clinical benefit observed in ICARS scores and functional abilities highlights the potential broader impact of the intervention on patients and caregivers [6]. In fact, higher ICARS scores have been shown to be correlated with higher Parental Distress domain scores [21].

Despite the requirement for weekly in‐hospital administration, treatment adherence remained high with few participants missing doses, suggesting that families found the benefits of therapy to outweigh the effort of weekly visits. Future trials could potentially include assessment of biomarkers that correlate with activity and clinical evaluations to evaluate benefits not captured by ICARS. Importantly, families reported improvements beyond those captured by ICARS, including better attention, communication, and overall functionality. Although not formally assessed in this study, these meaningful outcomes should be evaluated in future studies using appropriate methodologies.

Regarding the potential of liposomal M1P to reach the central nervous system, recent literature demonstrates that GLM101 treatment enhances expression levels of high‐mannose and complex/hybrid glycopeptides across numerous cellular proteins in individuals with PMM2 defects. Despite its relatively large molecular size and the anticipated limitation of crossing the blood–brain barrier, clinical improvements in central nervous system function have been observed in these patients. Whether GLM101's active ingredient M1P or its dephosphorylation product, mannose, crosses the blood–brain barrier was not assessed in this study. However, mannose transport across the blood–brain barrier by sugar transporters such as GLUT‐1 provides a plausible indirect mechanism for CNS exposure [23].

In conclusion, treatment with GLM101 was well tolerated and showed meaningful clinical improvements in ataxia and related functional domains in this short case series of patients with PMM2‐CDG. Taken together, the findings support further evaluation of GLM101 in coming clinical trials. Moreover, future studies should explore whether initiating therapy at earlier stages of the disease could be key not only to maximizing functional benefits but also to preserving cerebellar volume and preventing atrophy.

## Author Contributions

R.M., P.M., and M.S. participated in the conceptualization and methodology the study. F.E. and M.S. participated in the data acquisition. All authors participated in the investigation and formal analysis. R.M., P.M., and M.S. wrote the first draft of the report. All authors participated in drafting a significant portion of the manuscript or figures and in the writing of the manuscript. All authors had full access to all the data in the study, verified the data, and had final responsibility for the decision to submit for publication.

## Funding

F.E. is supported by grant FI22/00218, funded by Instituto de Salud Carlos III (ISCIII) and the European Union, NextGenerationEU, Mecanismo de Recuperación y Resiliencia (MRR). This study was funded by Glycomine Inc.

## Ethics Statement

The study was conducted in accordance with the ethical principles outlined in the Declaration of Helsinki and was consistent with the International Council for Harmonization Good Clinical Practice guidelines and applicable regulatory requirements on bioethics. Ethical permission for the study was obtained from the Research & Ethics Committee of the SJD Research Foundation and the AEMPS. All participants provided written informed consent for their participation in the Phase 2 study. When impossible due to intellectual disability, adult participants' guardians gave their written informed consent, and adolescents and adults gave their assent.

## Conflicts of Interest

M.S. and F.E. have nothing to report. R.M. and P.M. are Glycomine employees.

## Supporting information


**Table S1:** Item‐level ICARS scores (Baseline, Weeks 12 and 24).

**Data S1:** ICARS—raw data.

## Data Availability

The data that support the findings of this study are available on request from the corresponding author. The data are not publicly available due to privacy or ethical restrictions.
