# Supplementary material for: Results From a Phase 2, Open‐Label Study Evaluating the Safety, Tolerability, and Effect on Ataxia of GLM101 in Three Adult Patients With PMM2‐CDG
Source: J Inherit Metab Dis. 2026 Sep 7;49(5):e70240. doi: 10.1002/jimd.70240 (PMC13548179; doi:10.1002/jimd.70240)
Supplement: Supplementary file 1 [file JIMD-49-0-s001.docx]

| **ICARS Item Number** | **Potential Points** | **S01** | | | **S02** | | | **S03** | | |
| --- | --- | --- | --- | --- | --- | --- | --- | --- | --- | --- |
|  |  | Baseline | Week 12 | Week 24 | Baseline | Week 12 | Week 24 | Baseline | Week 12 | Week 24 |
| **1** | 8 | 3 | 3 | 3 | 7 | 6 | 6 | 3 | 2 | 2 |
| **2** | 4 | 2 | 2 | 1 | 4 | 4 | 4 | 2 | 1 | 1 |
| **3** | 6 | 2 | 2 | 2 | 4 | 4 | 4 | 1 | 2 | 2 |
| **4** | 4 | 1 | 1 | 1 | 4 | 2 | 2 | 1 | 0 | 0 |
| **5** | 4 | 2 | 2 | 1 | 4 | 4 | 4 | 1 | 1 | 1 |
| **6** | 4 | 2 | 2 | 2 | 4 | 4 | 4 | 2 | 2 | 1 |
| **7** | 4 | 1 | 0 | 0 | 2 | 0 | 0 | 1 | 0 | 0 |
| **Gait** | **34** | **13** | **12** | **10** | **29** | **24** | **24** | **11** | **8** | **7** |
| **8L / 8R** | 4 / 4 | 3 / 2 | 2 / 2 | 2 / 1 | 2 / 3 | 2 / 2 | 2 / 3 | *NV / NV* | *NV / NV* | *NV / NV* |
| **9L / 9R** | 4 / 4 | 3 / 3 | 0 / 0 | 1 / 1 | 3 / 3 | 1 / 1 | 1 / 0 | *NV / NV* | *NV / NV* | *NV / NV* |
| **10L / 10R** | 4 / 4 | 3 / 2 | 2 / 2 | 2 / 1 | 2 / 3 | 2 / 1 | 2 / 1 | 2 / 1 | 1 / 1 | 1 / 1 |
| **11L / 11R** | 4 / 4 | 2 / 2 | 1 / 1 | 1 / 1 | 2 / 2 | 2 / 1 | 2 / 1 | 2 / 1 | 2 / 1 | 1 / 0 |
| **12L / 12R** | 4 / 4 | 2 / 2 | 1 / 1 | 1 / 1 | 2 / 2 | 0 / 0 | 0 / 0 | 2 / 2 | 2 / 1 | 1 / 0 |
| **13R / 13L** | 4 / 4 | 3 / 3 | 2 / 3 | 3 / 2 | 3 / 3 | 3 / 3 | 3 / 3 | 2 / 2 | 2 / 2 | 1 / 1 |
| **14** | 4 | 2 | 2 | 2 | 2 | 1 | 1 | 1 | 1 | 1 |
| **Limb Coordination** | **52** | **32** | **19** | **19** | **32** | **19** | **19** | **15** | **13** | **7** |
| **15** | 4 | 2 | 1 | 2 | 3 | 2 | 1 | 2 | 1 | 1 |
| **16** | 4 | 2 | 2 | 2 | 2 | 2 | 2 | 1 | 1 | 0 |
| **Speech** | **8** | **4** | **3** | **4** | **5** | **4** | **3** | **3** | **2** | **1** |
| **17** | 3 | 2 | 1 | 1 | 2 | 1 | 1 | 2 | 2 | 1 |
| **18** | 2 | 1 | 1 | 1 | 1 | 1 | 1 | 0 | 0 | 0 |
| **19** | 1 | 0 | 0 | 0 | 0 | 0 | 0 | 0 | 0 | 0 |
| **Oculomotor** | **6** | **3** | **2** | **2** | **3** | **2** | **2** | **2** | **2** | **1** |
| **Total Score** | **100** | **52** | **36** | **35** | **69** | **49** | **48** | **31** | **25** | **16** |

## Supplemental Table 1. Item-level ICARS Scores (Baseline, Week 12, Week 24)

*ICARS assessments 8 and 9 for S03 were performed incorrectly at baseline (sitting instead of supine) and were therefore excluded from total ICARS scores for all subsequent visits; this affected the absolute scores but did not impact evaluation of change over time.
