# Supplementary material for: Results From a Phase 2, Open‐Label Study Evaluating the Safety, Tolerability, and Effect on Ataxia of GLM101 in Three Adult Patients With PMM2‐CDG
Source: J Inherit Metab Dis. 2026 Sep 7;49(5):e70240. doi: 10.1002/jimd.70240 (PMC13548179; doi:10.1002/jimd.70240)
Supplement: Supplementary file 2 [file JIMD-49-0-s002.docx]

Supplementary Data: ICARS - Raw Data

| Patient | S01 |  |  |  | S02 |  |  |  | S03 |  |  |  |
| --- | --- | --- | --- | --- | --- | --- | --- | --- | --- | --- | --- | --- |
| Subdomain | B | W12 | W24 | W28* | B | W12 | W24 | W28* | B | W12 | W24 | W28* |
| Posture & Gait | 13 | 12 | 10 | 11 | 29 | 24 | 24 | 25 | 11 | 8 | 7 | 7 |
| Limb Coordination | 32 | 19 | 19 | 25 | 32 | 19 | 19 | 21 | 15 | 13 | 7 | 7 |
| Speech | 4 | 3 | 4 | 4 | 5 | 4 | 3 | 3 | 3 | 2 | 1 | 1 |
| Oculomotor | 3 | 2 | 2 | 2 | 3 | 2 | 2 | 2 | 2 | 2 | 1 | 1 |
| Total ICARS | 52 | 36 | 35 | 42 | 69 | 49 | 48 | 51 | 31 | 25 | 16 | 16 |
| Change from B (Points) |  | -16 | -17 | -7 |  | -20 | -21 | 3 |  | -6 | -15 | 0 |

B: baseline, W: week.*Evaluation after withdrawal
